# NRLMF*β*: Beta-distribution-rescored neighborhood regularized logistic matrix factorization for improving the performance of drug–target interaction prediction

**DOI:** 10.1016/j.bbrep.2019.01.008

**Published:** 2019-02-07

**Authors:** Tomohiro Ban, Masahito Ohue, Yutaka Akiyama

**Affiliations:** a School of Computing, Tokyo Institute of Technology, 2-12-1 W8-76 Ookayama, Meguro-ku, Tokyo, 152-8550, Japan; b AIST-TokyoTech Real World Big-Data Computation Open Innovation Laboratory (RWBC-OIL), National Institute of Advanced Industrial Science and Technology, 1-1-1 Umezono, Tsukuba, Ibaraki, 305-8560, Japan; c Middle Molecule IT-based Drug Discovery Laboratory (MIDL), Tokyo Institute of Technology, RGBT2-A-1C 3-25-10 Tonomachi, Kawasaki-ku, Kawasaki City, Kanagawa, 210-0821, Japan; d Molecular Profiling Research Center for Drug Discovery (molprof), National Institute of Advanced Industrial Science and Technology, 2-4-7 Aomi, Koto-ku, Tokyo, 135-0064, Japan

**Keywords:** Drug–target interaction prediction, Neighborhood regularized logistic matrix factorization, Beta distribution, Rescoring, Bayesian optimization, Bayesian inference

## Abstract

Techniques for predicting interactions between a drug and a target (protein) are useful for strategic drug repositioning. Neighborhood regularized logistic matrix factorization (NRLMF) is one of the state-of-the-art drug–target interaction prediction methods; it is based on a statistical model using the Bernoulli distribution. However, the prediction is not accurate when drug–target interaction pairs have less interaction information (e.g., the sum of the number of ligands for a target and the number of target proteins for a drug). This study aimed to address this issue by proposing NRLMF with beta distribution rescoring (NRLMF*β*), which is an algorithm to improve the score of NRLMF. The score of NRLMF*β* is equivalent to the value of the original NRLMF score when the concentration of the beta distribution becomes infinity. The beta distribution is known as a conjugative prior distribution of the Bernoulli distribution and can reflect the amount of interaction information to its shape based on Bayesian inference. Therefore, in NRLMF*β*, the beta distribution was used for rescoring the NRLMF score. In the evaluation experiment, we measured the average values of area under the receiver operating characteristics and area under precision versus recall and the 95% confidence intervals. The performance of NRLMF*β* was found to be better than that of NRLMF in the four types of benchmark datasets. Thus, we concluded that NRLMF*β* improved the prediction accuracy of NRLMF. The source code is available at https://github.com/akiyamalab/NRLMFb.

## Highlights

• Strategic drug repositioning requires drug−target interaction prediction
• NRLMF does not provide accurate prediction with less interaction information
• Thus, we proposed NRLMF with beta distribution rescoring (NRLMF*β*)
• We found that NRLMF*β* improved the prediction accuracy of NRLMF

## Graphical abstract

**Figure:**
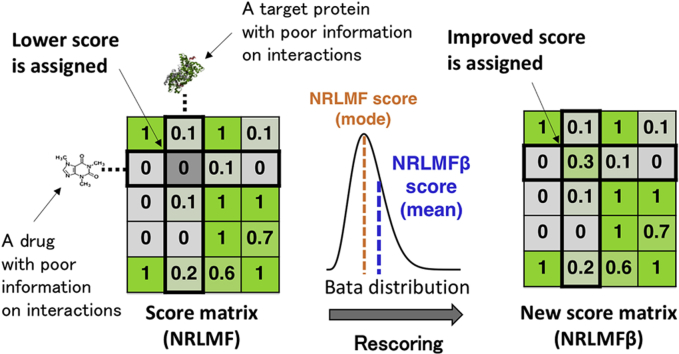

## 1 Introduction

Improving the accuracy for predicting drug–target interactions is an important task in drug discovery. Recently, research and development expenses are increasing annually, although the number of approvals for new drugs has remained constant every year [1]. Thus, increasing the value of existing drugs is necessary. Drug repositioning is one of the strategies that reuse an existing drug as a remedy for another disease [2]. For example, digoxin has been applied to prostate cancer in addition to heart failure therapy [3]. Previously, drug repositioning was casually performed during basic research and clinical trials [2]. However, it became more strategic with the development of machine learning and statistical models [4,5]. In particular, drug–target interaction prediction is known as one of the effective approaches [6,7].

Various methods have been proposed for predicting drug–target interactions [[7], [8], [9], [10], [11], [12], [13], [14], [15]]. These methods predict unknown interactions based on known interactions and the similarity of molecular structures (e.g., Tanimoto coefficient of chemical structures and normalized Smith–Waterman score of amino acids). At present, similar drugs are assumed to interact with similar proteins and similar proteins with similar drugs. These prediction methods can be divided into two approaches. One approach involves the use of kernel method, such as WNNGIP [8], BLMNII [9], LIK [10], and ECkNN [11], and the other involves the use of a matrix factorization model, such as MSCMF [14] and NRLMF [15]. To our knowledge, NRLMF is one of the most accurate methods.

However, matrix factorization models such as NRLMF cannot satisfactorily predict interactions when their number of related interactions is small. Two approaches are known to deal with this problem. One approach replaces the latent feature vectors of drugs or target proteins whose interaction number is 0 with weighted sums of latent vectors of most similar drugs or proteins [15,16]. The other adds a bias vector to each pair of drug and target protein [16]. However, with these methods, when a drug or target protein with high similarity does not exist for a drug or protein with a low number of interactions, an appropriate latent feature vector cannot be estimated. Therefore, we considered that directly correcting the score by using the number of interactions of each drug–target pair would be effective.

**Figure 1:**
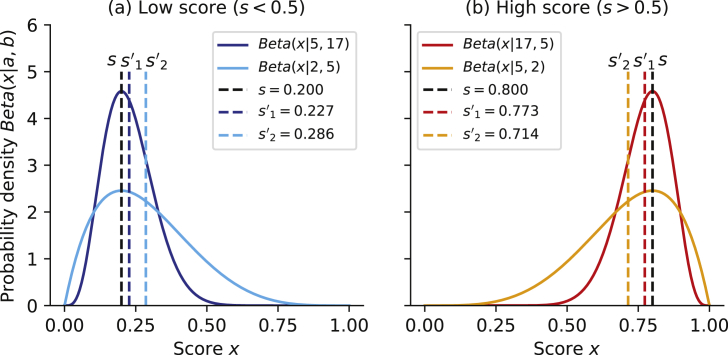
A plot showing the probability density function of the beta distribution and the improved value of the score. (a) Shows the change in the score when the original score *s* (assuming NRLMF score) is less than 0.5. $s{'}_{1}$ is the concentration defined by a+b (assuming the new feature *γ*) value, and $s{'}_{2}$ represents the score when the concentration value is small. Similarly, (b) shows the change in the score when the original score *s* is greater than 0.5.

In this study, we proposed NRLMF with beta distribution rescoring (NRLMF*β*), which is a new drug–target interaction prediction model based on rescoring of the NRLMF score. Since NRLMF is based on the Bernoulli distribution, the beta distribution, which is its conjugate prior distribution, is used (Fig. 1). Our findings might form the basis to improve the accuracy of drug–target interaction prediction models. Herein, we introduce a new feature quantity to define the amount of interaction information and describe how to rescore the algorithm. Further, in order to confirm the improvement of prediction accuracy, we conducted a comparison experiment between the proposed method NRLMF*β* and the conventional method NRLMF. In the experiment, we evaluated the area under the receiver operating characteristics (AUC) and area under precision versus recall (AUPR) by using the general benchmark [7].

## 2 Materials and methods

### 2.1 Materials

Evaluation experiments were performed using a benchmark dataset [7], which is generally used in drug–target interaction prediction [[7], [8], [9],^12^,^14^,^15^]. The benchmark consists of four datasets targeting proteins of Nuclear Receptor, GPCR, Ion Channel, and Enzyme. Each dataset includes three matrices—an interaction matrix, a drug similarity matrix, and a protein similarity matrix. The interaction matrix is defined by an adjacency matrix consisting of 0 and 1; it takes a value of 1, if interaction is experimentally confirmed between the drug and target protein, otherwise it takes a value of 0. The interaction information was obtained from the KEGG BRITE [17], BRENDA [18], SuperTarget [19], and DrugBank [20] databases. The statistical information of the interaction matrix in each dataset is shown in Table 1. The similarity matrix of a drug is defined by a real matrix taking a value ranging from 0 to 1. The chemical structures are considered to be similar, if the value is close to 1. The structure information of drugs was obtained from KEGG LIGAND [17] database. Notably, drugs having molecular weight less than 100 were excluded in order to exclude factors such as ions and cofactors. Tanimoto coefficient (i.e., $S_{drug}\left(d,d'\right)=|d\cap d'|/|d\cup d'|$ for drugs *d* and d', calculated by SIMCOMP [21]) was used for calculating the similarity between drugs. The similarity matrix of target proteins was also defined as a real matrix that takes values from 0 to 1. Amino acid sequences obtained from KEGG GENES [17] database were used for the target proteins. The similarity between target proteins was calculated using the normalized Smith–Waterman score (calculated using $S_{target}\left(t,t'\right)=SW\left(t,t'\right)/\sqrt{SW\left(t,t\right)SW\left(t',t'\right)}$ for targets t,t'). Here, SW(⋅,⋅) represents the Smith–Waterman score [22]. These datasets can be obtained at http://web.kuicr.kyoto-u.ac.jp/supp/yoshi/drugtarget/.

**Table 1:** Statistics for the drug–target interaction datasets.

Statistics	Nuclear receptor	GPCR	Ion channel	Enzyme
No. of drugs	54	223	210	445
No. of targets	26	95	204	664
No. of interactions	90	635	1476	2926

### 2.2 Problem formalization

Herein, we denote the set of drugs as $D={\left\{d_{i}\right\}}_{i=1}^{n_{d}}$, and the set of targets as $T={\left\{t_{j}\right\}}_{j=1}^{n_{t}}$, where $n_{d}$ and $n_{t}$ are the number of elements in sets D and T, respectively. The interaction matrix is represented by the adjacency matrix $Y\in {\left\{0,1\right\}}^{n_{d}\times n_{t}}$. In the matrix Y, the value of drug $d_{i}$ and target $t_{j}$ is expressed as $y_{ij}\in \left\{0,1\right\}$. Thus, let $y_{ij}=1$ (interaction pair), if interaction is observed, or let $y_{ij}=0$ (unknown pair). In the interaction matrix, drugs that do not show interaction with any targets are expressed as $D^{-}=\left\{d_{i}\in D|\forall l,y_{il}=0\right\}$ (negative drugs), whereas those that have one or more interactions are denoted as $D^{+}=D\backslash D^{-}$ (positive drugs). Similarly, targets that do not have interaction with any drugs are expressed as $T^{-}=\left\{t_{j}\in T|\forall l,y_{lj}=0\right\}$ (negative targets), whereas those that have one or more interactions are denoted as $T^{+}=T\backslash T^{-}$ (positive targets). The drug similarity matrix is expressed as $S_{d}\in {\left[0,1\right]}^{n_{d}\times n_{d}}$, and the similarity between drug $d_{i}$ and drug $d_{l}$ is denoted as ${\left(S_{d}\right)}_{il}\in \left[0,1\right]$. Similarly, the target similarity matrix is expressed as $S_{t}\in {\left[0,1\right]}^{n_{t}\times n_{t}}$, and the similarity between target $t_{j}$ and target $t_{l}$ is denoted as ${\left(S_{t}\right)}_{jl}\in \left[0,1\right]$. This problem intends to assign high scores for drug–target pairs that have a possibility of interaction in unknown pairs.

### 2.3 NRLMF

NRLMF [15] is one of the drug–target prediction methods based on a matrix factorization technique and is one of the state-of-the-art methods.

#### 2.3.1 Interaction probability

The possibility that a drug $d_{i}$ and target $t_{j}$ interact is evaluated by the interaction probability $p_{ij}$ calculated from latent feature vectors of the drug and target. The latent feature vector of drug $d_{i}$ is represented by $u_{i}\in {\mathbb{R}}^{r}$, and that of the target protein $t_{j}$ is represented by $v_{j}\in {\mathbb{R}}^{r}$, where *r* is a hyperparameter representing the number of dimensions of the latent feature vector. $U\in {\mathbb{R}}^{n_{d}\times r}$ is a latent matrix that has the latent feature vector $u_{i}^{⊤}$ as a row vector, and $V\in {\mathbb{R}}^{n_{t}\times r}$ is a latent feature matrix that has the latent feature vector $v_{j}^{⊤}$ as a row vector. Thus, the interaction probability between drug $d_{i}$ and target $t_{j}$ is defined as follows:

(1) $$p_{ij}=\frac{\text{exp}\left(u_{i}^{⊤}v_{j}\right)}{1+\text{exp}\left(u_{i}^{⊤}v_{j}\right)}.$$

#### 2.3.2 Prediction model

By using the interaction probability $p_{ij}$, we defined the likelihood of interaction matrix Y in the latent feature matrix U,V as follows:

(2) $$\text{Pr}\left(Y|U,V,c\right)=\overset{n_{d}}{\underset{i=1}{\prod }}\overset{n_{t}}{\underset{j=1}{\prod }}p_{ij}^{cY_{ij}}{\left(1-p_{ij}\right)}^{1-Y_{ij}},$$

where c>0 is a hyperparameter. Here, we assumed that the latent feature vector $u_{i}$ follows a multivariate normal distribution with mean $0\in {\mathbb{R}}^{r}$ and variance-covariance matrix $\lambda_{d}^{-1}I\in {\mathbb{R}}^{r\times r}$, and then the latent feature matrix U follows the joint distribution defined as equation (3). Similarly, we assumed that the latent feature vector $v_{j}$ follows a multivariate normal distribution with mean $0\in {\mathbb{R}}^{r}$ and variance-covariance matrix $\lambda_{t}^{-1}I\in {\mathbb{R}}^{r\times r}$, and then the latent feature matrix V follows the joint distribution defined as equation (4). Where $\lambda_{d},\lambda_{t}>0$ are hyperparameters, and $I\in {\mathbb{R}}^{r\times r}$ is an identity matrix.

(3) $$\text{Pr}\left(U|\lambda_{d}\right)=\overset{n_{d}}{\underset{i=1}{\prod }}N\left(u_{i}|0,\lambda_{d}^{-1}I\right),$$

(4) $$\text{Pr}\left(V|\lambda_{t}\right)=\overset{n_{t}}{\underset{j=1}{\prod }}N\left(v_{j}|0,\lambda_{t}^{-1}I\right),$$

From equations (2), (3), (4), we can obtain a solution of the maximum a posteriori (MAP) by solving the following minimization problem:

(5) $$\underset{U,V}{\text{min}}\overset{n_{d}}{\underset{i=1}{\sum }}\overset{n_{t}}{\underset{j=1}{\sum }}\left(1+cy_{ij}-y_{ij}\right)\text{ln}\left[1+\text{exp}\left(u_{i}^{⊤}v_{j}\right)\right]-cy_{ij}u_{i}^{⊤}v_{j}+\frac{\lambda_{d}}{2}{\left\|U\right\|}_{F}^{2}+\frac{\lambda_{t}}{2}{\left\|V\right\|}_{F}^{2}$$

where $∥\cdot {∥}_{F}$ is the Frobenius norm.

#### 2.3.3 Regularization

We introduced a regularization term to bring the latent feature vectors of high similarity drugs or targets close to each other by using the similarity matrices $S_{d}$ and $S_{t}$. The regularization term in the latent feature vector of the drug is defined using the following equation.

(6) $$\overset{n_{d}}{\underset{i=1}{\sum }}\overset{n_{t}}{\underset{l=1}{\sum }}a_{il}{\left\|u_{i}-u_{l}\right\|}_{2}^{2}=tr\left(U^{⊤}L_{d}U\right)$$

(7) $$a_{il}=\left\{ \begin{array}{cc} {\left(S_{d}\right)}_{il} & \mathrm{if}\;d_{l}\in NN_{K_{1}}\left(d_{i}\right)\\ 0 & otherwise \end{array}\right.$$

where $L_{d}=\left(D^{d}+{\tilde{D}}^{d}\right)-\left(A+A^{⊤}\right)\in {\mathbb{R}}^{n_{d}\times n_{d}}$ is a real matrix. $D^{d}$ and ${\tilde{D}}^{d}$ are two diagonal matrices, in which the diagonal elements are $D_{ii}^{d}={\sum^{\text{​}}}_{l=1}^{n_{d}}a_{il}$ and ${\tilde{D}}_{ll}^{d}={\sum^{\text{​}}}_{i=1}^{n_{d}}a_{il}$. $A\in {\mathbb{R}}^{n_{d}\times n_{d}}$ is a matrix in which the element is $a_{il}$ in equation (7). Conversely, $NN_{K_{1}}\left(d_{i}\right)$ is a $K_{1}$ nearest neighbor decided by the *i*-th row vector in the matrix $S_{d}$, where $K_{1}\in \mathbb{N}$ is a hyperparameter. In addition, $∥\cdot {∥}_{2}$ is the Euclidean norm, and tr(⋅) is the trace function. Similarly, the regularization term in the latent feature vector of the target is defined using the following equation.

(8) $$\overset{n_{t}}{\underset{j=1}{\sum }}\overset{n_{t}}{\underset{l=1}{\sum }}b_{jl}{\left\|v_{j}-v_{l}\right\|}_{2}^{2}=tr\left(V^{⊤}L_{t}V\right)$$

(9) $$b_{jl}=\left\{ \begin{array}{cc} {\left(S_{t}\right)}_{jl} & \mathrm{if}\;t_{l}\in NN_{K_{1}}\left(t_{j}\right)\\ 0 & otherwise \end{array}\right.$$

where $L_{t}=\left(D^{t}+{\tilde{D}}^{t}\right)-\left(B+B^{⊤}\right)\in {\mathbb{R}}^{n_{t}\times n_{t}}$ is a real matrix. $D^{t}$ and ${\tilde{D}}^{t}$ are two diagonal matrices, in which the diagonal elements are $D_{jj}^{t}={\sum^{\text{​}}}_{l=1}^{n_{t}}b_{jl}$ and ${\tilde{D}}_{ll}^{t}={\sum^{\text{​}}}_{j=1}^{n_{t}}b_{jl}$. $B\in {\mathbb{R}}^{n_{t}\times n_{t}}$ is a matrix in which the element is $b_{il}$ in equation (9).

#### 2.3.4 Estimation and scoring

By introducing the regularization terms (6) and (8) into (5), we redefined the minimization problem as follows:

(10) $$\begin{array}{cc} \underset{U,V}{\text{min}}\overset{n_{d}}{\underset{i=1}{\sum }}\overset{n_{t}}{\underset{j=1}{\sum }} & \left(1+cy_{ij}-y_{ij}\right)\text{ln}\left[1+\text{exp}\left(u_{i}^{⊤}v_{j}\right)\right]-cy_{ij}u_{i}^{⊤}v_{j}\\ & +\frac{1}{2}tr\left[U^{⊤}\left(\lambda_{d}I_{d}+\alpha L_{d}\right)U\right]+\frac{1}{2}tr\left[V^{⊤}\left(\lambda_{t}I_{t}+\beta L_{t}\right)V\right] \end{array}$$

where $I_{d}\in {\mathbb{R}}^{n_{d}\times n_{d}}$ and $I_{t}\in {\mathbb{R}}^{n_{t}\times n_{t}}$ are identity matrices, and α,β>0 are hyperparameters. Alternating gradient descent method [23] was used for optimizing equation (10). In addition, AdaGrad algorithm [24] that has a hyperparameter θ>0 corresponding to the gradient coefficient was used to accelerate the speed of optimization. The row vectors corresponding to negative drugs and targets for the MAP solution obtained using the optimization were modified. For a negative drug $d_{i}\in D^{-}$, $K_{2}$ nearest neighbors of the positive drugs are denoted as $NN_{K_{2}}\left(d_{i}\right)\subset D^{+}$, where $NN_{K_{2}}\left(d_{i}\right)$ is determined like $NN_{K_{1}}\left(d_{i}\right)$ except that it targets positive drugs. Here, the latent feature vector ${\hat{u}}_{i}\in {\mathbb{R}}^{r}$ of the drug $d_{i}$ corresponding to the *i*-th row vector of the latent feature matrix $\hat{U}$ is modified as a vector ${\tilde{u}}_{i}\in {\mathbb{R}}^{r}$ by using the following equation.

(11) $${\tilde{u}}_{i}=\{\begin{array}{cc} {\hat{u}}_{i} & \text{if}\;d_{i}\in D^{+}\\ \frac{\sum_{d_{l}\in NN_{K_{2}}\left(d_{i}\right)}{\left(S_{d}\right)}_{il}{\hat{u}}_{l}}{\sum_{d_{l}\in NN_{K_{2}}\left(d_{i}\right)}{\left(S_{d}\right)}_{il}} & \text{if}\;d_{i}\in D^{-} \end{array}$$

Likewise, negative target $t_{j}\in T^{-}$ is also modified. Thus, by using the modified latent feature vectors ${\tilde{u}}_{i},{\tilde{v}}_{j}\in {\mathbb{R}}^{r}$, we calculated the score of drug $d_{i}\in D$ and target $t_{j}\in T$ by using the following equation.

(12) $$s_{ij}=\frac{\text{exp}\left({\tilde{u}}_{i}^{⊤}{\tilde{v}}_{j}\right)}{1+\text{exp}\left({\tilde{u}}_{i}^{⊤}{\tilde{v}}_{j}\right)}.$$

### 2.4 NRLMF*β*

NRLMF*β* is an algorithm that rescores the score of NRLMF as the expected value of the beta distribution, which is determined based on interaction information and NRLMF score. The beta distribution is known as a conjugative prior distribution of the Bernoulli distribution used in NRLMF and can reflect the amount of interaction information to NRLMF*β* score.

#### 2.4.1 New feature quantity

We defined a new feature quantity $\gamma_{ij}\in {\mathbb{Z}}_{+}$ (where ${\mathbb{Z}}_{+}$ is expressed as a set of integers greater than or equal to 0) as a quantity of the interaction information for any drug $d_{i}$ and target $t_{j}$ pair by using the following equation.

(13) $$\gamma_{ij}=\overset{n_{t}}{\underset{l=1}{\sum }}\;y_{il}+\overset{n_{d}}{\underset{l'=1}{\sum }}\;y_{l'j}$$

The feature quantity $\gamma_{ij}$ represents the sum of the observed values of the *i*-th row and in the *j*-th column in the interaction matrix Y. The $\gamma_{ij}$ has a greater value if the number of interaction pairs in the *i*-th row and *j*-th column become larger.

#### 2.4.2 Re-scoring using the beta distribution

The NRLMF score $s_{ij}$ based on the value of the feature quantity $\gamma_{ij}$ becomes too small when there is little information on the interaction. To address this, we performed rescoring for the score of drug $d_{i}$ and target $t_{j}$ by using the beta distribution defined using the following equation.

(14) $$Beta\left(x|a_{ij},b_{ij}\right)=\frac{x^{a_{ij}-1}{\left(1-x\right)}^{b_{ij}-1}}{B\left(a_{ij},b_{ij}\right)}$$

where $a_{ij},b_{ij}>0$ are parameters for determining the shape of the beta distribution, and the condition of $a_{ij},b_{ij}>1$ was assumed in order to limit the distribution to bell shape. B(⋅,⋅) is a beta function. We defined the relationship between parameters $a_{ij},b_{ij}$, and score $s_{ij}$ and the feature $\gamma_{ij}$ by using the following equation.

(15) $$\frac{a_{ij}-1}{a_{ij}+b_{ij}-2}=s_{ij}$$

(16) $$a_{ij}+b_{ij}=\gamma_{ij}\cdot \eta_{1}+\eta_{2}$$

where $\eta_{1},\eta_{2}>0$ are hyperparameters, and $\eta_{2}>2$ from the above-mentioned conditions refer to the shape of the distribution. In the above equations, equation (15) refers to the mode of the beta distribution, and equation (16) refers to the concentration. Notably, the score of NRLMF corresponds to the mode of the beta distribution. From these relational expressions, the score $s{'}_{ij}$ of NRLMF*β* is defined as the expected value of the beta distribution by using the following equation.

(17) $$s{'}_{ij}=\frac{a_{ij}}{a_{ij}+b_{ij}}=\frac{s_{ij}\cdot \left(\gamma_{ij}\cdot \eta_{1}+\eta_{2}-2\right)+1}{\gamma_{ij}\cdot \eta_{1}+\eta_{2}}$$

The score $s{'}_{ij}$ of NRLMF*β* is equivalent to the NRLMF score $s_{ij}$ when the concentration of the beta distribution becomes infinity. In order to prove the argument, we assume that $\eta_{1}$ is at infinity and $\eta_{2}$ is any finite value. Then, the argument is proved using equation (18).

(18) $$\underset{\eta_{1}\to \infty }{\text{lim}}s{'}_{ij}=\underset{\eta_{1}\to \infty }{\text{lim}}\frac{s_{ij}\cdot \left(\gamma_{ij}+\frac{\eta_{2}}{\eta_{1}}-\frac{2}{\eta_{1}}\right)+\frac{1}{\eta_{1}}}{\gamma_{ij}+\frac{\eta_{2}}{\eta_{1}}}=\frac{s_{ij}\cdot \gamma_{ij}}{\gamma_{ij}}=s_{ij}$$

#### 2.4.3 Algorithm

The calculation procedure of NRLMF*β* is shown in Algorithm 1. Initially, the observed value $Y\in {\left\{0,1\right\}}^{n_{d}\times n_{t}}$ of the interaction matrix and the similarity matrices $S_{d},S_{t}$ are given as inputs. Next, the values of hyperparameters $c,K_{1},K_{2},r,\lambda_{d},\lambda_{t},\alpha ,\beta ,\theta ,\eta_{1},\eta_{2}$ are set, and the NRLMF score $s_{ij}$ is calculated for arbitrary *i*, *j* based on equation (12). For each *i* and *j*, $\gamma_{ij}$ is calculated from the observed value Y by using equation (13). From the calculated $\gamma_{ij}$ and NRLMF score $s_{ij}$, the NRLMF*β* score $s{'}_{ij}$ is calculated using equation (17). This algorithm rescores the score of NRLMF for all drug and protein pairs in the interaction matrix.

**Figure:**
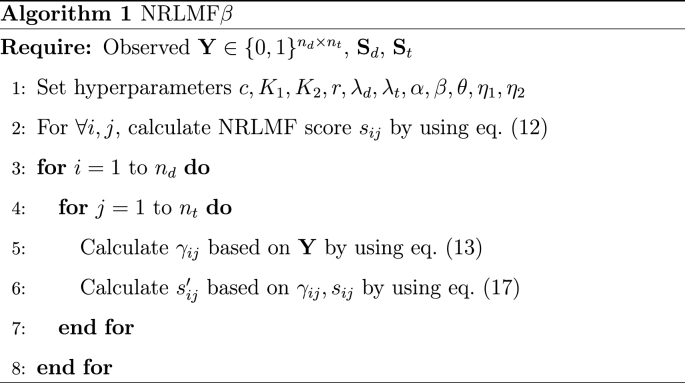

### 2.5 Experimental settings

In order to compare the generalization performance of NRLMF and NRLMF*β*, we used the AUC and AUPR as the evaluation index; according to Liu et al. [15], three kinds of 10-fold cross-validation scenarios (CVSs) called CVS1, CVS2, and CVS3 were performed five times each. The division of test data and training data is exactly the same as the comparison method NRLMF, because this evaluation result uses the script used in the original paper of the comparative method and uses the same random seed. In CVS1, all drug–target pairs $P_{1}=\left\{\left(d_{i},t_{j}\right)\in D\times T\right\}$ contained in the interaction matrix Y were randomly divided into ten sets so that the number of elements remained equal. A combination of one set ${P'}_{1}\subset P_{1}$ and observed values of the pair included therein were determined as the test data $\left\{\left(\left(d_{i},t_{j}\right),y_{ij}\right)|\left(d_{i},t_{j}\right)\in P{'}_{1}\right\}$. Further, an interaction matrix obtained by replacing all of the measured values $y_{ij}$ of pairs included in the test data with 0 is defined as training data $Y'\in {\left\{0,1\right\}}^{n_{d}\times n_{t}}$ ($Y{'}_{ij}=0$ for $\forall \left(d_{i},t_{j}\right)\in P{'}_{1}$, $Y{'}_{ij}=Y_{ij}$ for $\forall \left(d_{i},t_{j}\right)\notin P{'}_{1}$). Next, each of the 10 divided sets was treated repetitively as test data, and the values of the evaluation index AUC and AUPR were measured. The above operation was repeated 5 times, and the average value of each evaluation index and the 95% confidence interval of the *t*-distribution were calculated. Conversely, in CVS2, the set D of drugs was randomly divided into ten sets so that the number of elements was even. For a set of pairs $P{'}_{2}=\left\{\left(d_{i},t_{j}\right)\in D'\times T\right\}$ consisting of a combination of one divided D'⊂D and all of the targets T, a set of combinations of a pair included in the set and its observed value was determined as test data $\left\{\left(\left(d_{i},t_{j}\right),y_{ij}\right)|\left(d_{i},t_{j}\right)\in P{'}_{2}\right\}$. Next, like the CVS1, training data were prepared, and the value of the evaluation index was measured. Similarly, in CVS3, a set T of targets was randomly divided into ten sets so that the number of elements was even. A combination of a pair included in the set and the observed value was determined as test data $\left\{\left(\left(d_{i},t_{j}\right),y_{ij}\right)|\left(d_{i},t_{j}\right)\in P{'}_{3}\right\}$ for a pair set $P{'}_{3}=\left\{\left(d_{i},t_{j}\right)\in D\times T'\right\}$ consisting of a combination of one divided T' and all drug D, and then the values of the evaluation indexes were measured.

### 2.6 Hyperparameter optimization

In terms of the computational experiment, we cited the result of NRLMF from the original paper [15] and measured the performance of NRLMF*β*. Regarding the search range of hyperparameter of NRLMF*β*, based on [15], we defined the one common to NRLMF as $c=5,K_{1}=5,K_{2}=5,r=\left\{50,100\right\},\alpha =\left\{2^{-5},2^{-4},\ldots ,2^{2}\right\},\beta =\left\{2^{-5},2^{-4},\ldots ,2^{0}\right\},\lambda_{d}=\lambda_{t}=\left\{2^{-5},2^{-4},\ldots ,2^{1}\right\}$, and $\theta =\left\{2^{-3},2^{-2},\ldots ,2^{0}\right\}$. Conversely, the newly introduced hyperparameters $\eta_{1},\eta_{2}$ were defined as $\eta_{1}=\left\{2^{5},2^{6},\ldots ,2^{9}\right\},\eta_{2}=\left\{2^{2},2^{3},\ldots ,2^{5}\right\}$. Regarding optimization of hyperparameters, grid search and Bayesian optimization were combined for all datasets. This is because previous experimental results [25] showed that Bayesian optimization can perform the same evaluation as a grid search. Furthermore, by dividing the search range of the hyperparameter, we can simultaneously conduct Bayesian optimization, and the calculation time can be shortened. Therefore, the value of $r,\beta ,\theta ,\eta_{1},\eta_{2}$ was sequentially fixed based on the search range defined above, and Bayesian optimization was performed on the search range of the remaining $\lambda_{d}$ and *α* ($\lambda_{t}$ is equal to $\lambda_{d}$). Next, among solutions obtained using Bayesian optimization, those with the highest AUC average value were selected as optimal hyperparameters (Table S1). Here, we used the Gaussian process mutual information algorithm [26] as the Bayesian optimization method, and the parameter *δ* was $10^{-100}$ according to Ref. [25].

These calculations were performed using supercomputer TSUBAME 3.0 at the Tokyo Institute of Technology. Two Intel Xeon E5-2680 v4 (2.4 GHz, 14 cores) CPUs and 256 GB main memory were installed in the computing node, and calculation was performed simultaneously by using Shared Memory Parallel with seven cores (the maximum number of the q_node option on TSUBAME 3.0).

## 3 Results

By comparing the prediction accuracy (i.e., AUC and AUPR) of NRLMF and NRLMF*β*, we showed that performance is improved by rescoring by using the beta distribution.

### 3.1 Performance based on AUC

The average value of AUC calculated for each CVS for each dataset and the 95% confidence interval obtained using the *t*-distribution in order to compare the prediction accuracy of NRLMF and NRLMF*β* are shown in Table 2. For CVS1 and CVS3, the average value of AUC of NRLMF*β* was higher than that of NRLMF in all datasets. As for the dataset trend, both CVS1 and CVS3 were found to be remarkably improved since the dataset was smaller, indicating that NRLMF*β* exerts a large effect when the amount of data is small. In particular, for the nuclear receptor dataset of CVS3, the average value of AUC of NLRMF was 0.851, whereas that of NRLMF*β* was 0.941; an increase of 0.090 was observed. We will discuss this improvement in section 4.3. Conversely, for CVS2, the average value of AUC of NRLMF*β* was higher than that of NRLMF for datasets other than Enzyme. However, for Enzyme, the average value of AUC of NRLMF was 0.871, whereas that of NRLMF*β* was 0.858, i.e., the prediction accuracy decreased.

**Table 2:** The AUC for the 5×10-fold cross-validation scenarios.

Dataset	CVS1		CVS2		CVS3	
	NRLMF	NRLMF*β*	NRLMF	NRLMF*β*	NRLMF	NRLMF*β*
Nuclear receptor	0.950±0.011	0.964±0.007	0.900±0.021	0.906±0.022	0.851±0.027	0.941±0.021
GPCR	0.969±0.004	0.975±0.003	0.895±0.011	0.901±0.012	0.930±0.012	0.959±0.006
Ion channel	0.989±0.001	0.990±0.001	0.813±0.027	0.816±0.027	0.964±0.007	0.970±0.006
Enzyme	0.987±0.001	0.990±0.001	0.871±0.017	0.858±0.019	0.966±0.005	0.978±0.003

A 10-fold cross-validation was performed five times, and the average value of AUC and the 95% confidence interval of the *t*-distribution are shown. The one with higher average value is shown in bold.

### 3.2 Performance based on AUPR

The average value of AUPR calculated for each CVS for each dataset and the 95% confidence interval by the *t*-distribution in order to compare the prediction accuracy of NRLMF and NRLMF*β* are shown in Table 3. For CVS1 and CVS3, the average value of AUPR of NRLMF*β* was higher than that of NRLMF in all datasets. As for the trend of each dataset, since the dataset was smaller, the trend improved remarkably as in the case of AUC. In particular, for the nuclear receptor dataset of CVS3, the average value of AUPR of NRLMF was 0.449, whereas that of NRLMF*β* was 0.661; an increase of 0.212 was observed. Conversely, for CVS2, the average value of AUPR of NRLMF*β* was higher than that of NRLMF for datasets other than GPCR. For GPCR, the average value of AUPR of NRLMF was 0.364, whereas that of NRLMF*β* was 0.358, i.e., the prediction accuracy declined.

**Table 3:** The AUPR for the 5×10-fold cross-validation scenarios.

Dataset	CVS1		CVS2		CVS3	
	NRLMF	NRLMF*β*	NRLMF	NRLMF*β*	NRLMF	NRLMF*β*
Nuclear receptor	0.728±0.041	0.755±0.035	0.545±0.054	0.555±0.061	0.449±0.079	0.661±0.073
GPCR	0.749±0.015	0.755±0.014	0.364±0.023	0.358±0.024	0.556±0.038	0.572±0.039
Ion channel	0.906±0.008	0.913±0.008	0.344±0.033	0.346±0.033	0.785±0.028	0.798±0.027
Enzyme	0.892±0.006	0.897±0.006	0.358±0.040	0.360±0.038	0.812±0.018	0.815±0.018

A 10-fold cross-validation was performed five times, and the average value of AUPR and the 95% confidence interval of the *t*-distribution are shown. The one with higher average value is shown in bold.

## 4 Discussion

We investigated the search range of the newly introduced hyperparameters $\eta_{1},\eta_{2}$ and the reason why the performance of NRLMF*β* was better than that of NRLMF.

### 4.1 Performance based on external validation

In order to evaluate the generalization performance of the proposed method NRLMF*β*, we compared it with the conventional method NRLMF by external validation (nested cross-validation). In the external validation, in order to determine the optimal hyperparameter, 5-fold cross-validation by CVS1 was performed using the training data. Next, the hyperparameter that maximizes the average AUC was determined using Bayesian optimization [25]. Subsequently, AUC and AUPR were measured using the test data by using the determined hyperparameter. In this external validation, five times of 10-fold cross-validation (CVS1) were performed using the GPCR dataset used for the cross-validation. As a result, regarding AUC, NRLMF*β* was 0.974±0.003, which was higher than that for NRLMF 0.968±0.004. Further, for AUPR, NRLMF*β* was 0.759±0.016, exceeding that of NRLMF 0.751±0.015. These results indicate that generalization performance can be obtained even if only amino acid sequences not including a three-dimensional structure are used. If information of the three-dimensional structures is used, improving the generalization performance would be possible.

### 4.2 Characteristics of hyperparameters $\eta_{1},\eta_{2}$

**Figure 2:**
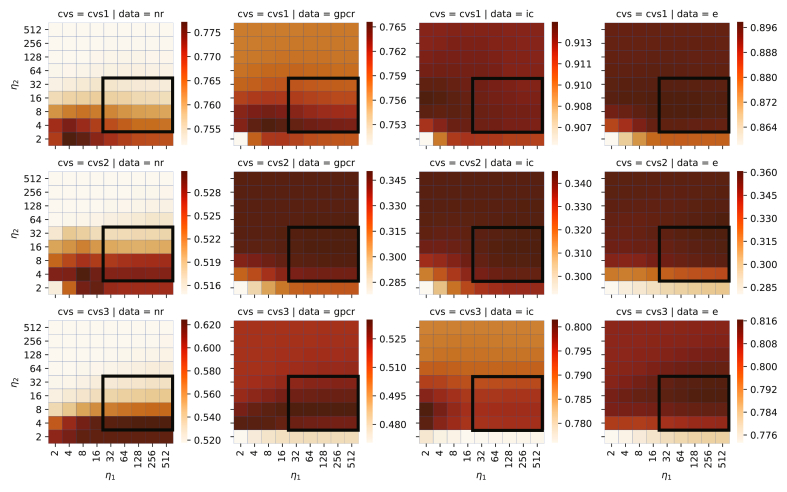
Heatmaps of AUPR when hyperparameters $\eta_{1},\eta_{2}$ are changed in each pair of cross-validation scenario (CVS) and dataset. The AUPR of each heatmap uses the average value of AUPR calculated for each CVS. However, hyperparameters without $\eta_{1},\eta_{2}$ were fixed using different values for each pair of CVS and dataset. In addition, the frame in each figure is the search range of the hyperparameters $\eta_{1},\eta_{2}$ defined in section 2.6.

The heatmap for the average value of AUPR when hyperparameters $\eta_{1},\eta_{2}$ of NRLMF*β* were changed from 2 to 512 for each pair of CVS and dataset, respectively, is shown in Fig. 2. However, for hyperparameters other than $\eta_{1},\eta_{2}$, CVS and dataset with the optimal solution were used to perform grid search with fixed $\eta_{1}=7$ and $\eta_{2}=3$ (see also Supplemental Information Fig. S1) by using different values for each pair of the group. The frame in each figure is the search range of the hyperparameters $\eta_{1},\eta_{2}$ defined in section 2.6. This frame was created based on a heatmap that was an average of 12 heatmaps shown in Fig. 2. Based on the balance between the calculation time and prediction accuracy, we found that the total value inside the frame of size 4×5 (where the section width of $\eta_{1}$ is 5 and that of $\eta_{2}$ is 4) was the largest. Thus, the frame area is $\eta_{1}=\left\{2^{5},2^{6},\ldots ,2^{9}\right\}$ and $\eta_{2}=\left\{2^{2},2^{3},\ldots ,2^{5}\right\}$. From Fig. 2, for nuclear receptor dataset, the average value of AUPR was found to fluctuate remarkably depending on the values of $\eta_{1},\eta_{2}$. This is because the size of the dataset was small; the change in the score value for the interaction pair was thought to remarkably influence the change in the value of AUPR. Therefore, since the size of the dataset increases with GPCR, Ion channel, and Enzyme, the change in the value of AUPR with respect to the change of $\eta_{1},\eta_{2}$ seemed to be small.

### 4.3 Characteristics of rescoring

A plot depicting the probability density function of the beta distribution and the improved value of the score in order to explain how the score of NRLMF changes by rescoring of NRLMF*β* is shown in Fig. 1. The score after improvement by rescoring of NRLMF*β* differs between when the score *s* before improvement is low (Low score: s<0.5) and when it is high (High score: s>0.5). The change when the score before improvement is low (Low score) and the change when the concentration is different was compared (Fig. 1(a)). When the original score *s* was 0.200, the improved score $s{'}_{1}$ of concentration 22 (a=5,b=17) was 0.227, and the improved score $s{'}_{2}$ of concentration 7 (a=2,b=5) was 0.286. As described above, when the score *s* before improvement was less than 0.5, the score after improvement tended to increase; as the concentration decreases, the amount increases. Conversely, Fig. 1(b) shows the change when the score before improvement is high (High score), and the change with different concentrations is compared. When the original score *s* was 0.800, the improved score $s{'}_{1}$ of concentration 22 (a=17,b=5) was 0.773, and the improved score $s{'}_{2}$ of concentration 7 (a=5,b=2) was 0.714. Thus, when the score *s* before improvement was larger than 0.5, the score after improvement decreased unlike that when the score before improvement was less than 0.5; when the concentration was further decreased, the amount further decreases.

### 4.4 Discussion about improvement

**Figure 3:**
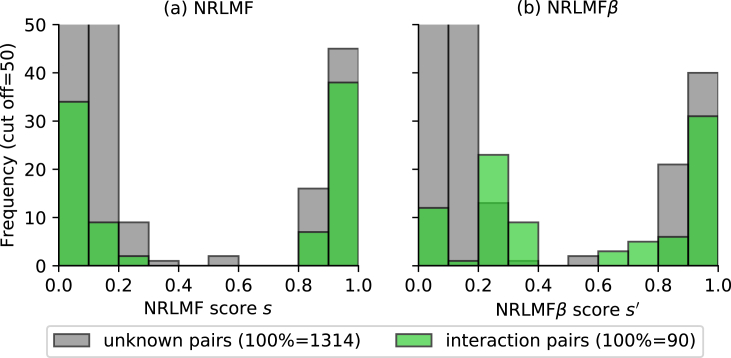
Scatter plot of scores s,s' and feature quantities *γ* of NRLMF and NRLMF*β*. The score was calculated using the leave-one-out method corresponding to CVS3 for the nuclear receptor dataset. The horizontal axis represents the score of each method, and the vertical axis represents the feature quantity we introduced.

**Figure 4:**
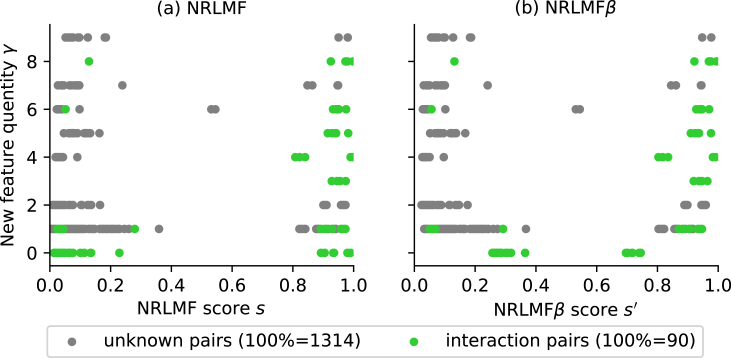
Histogram for NRLMF and NRLMF*β* scores s,s'. The score was calculated using the leave-one-out method corresponding to CVS3 for the nuclear receptor dataset. The horizontal axis represents the score of each method, and the vertical axis represents frequency (cutoff = 50).

A scatter plot of scores and the feature quantity *γ* in NRLMF and NRLMF*β* in order to assess the evaluation results of CVS3 for nuclear receptor dataset are shown in Fig. 3. Here, the scores s,s' and feature quantity *γ* of each method were calculated by substituting the leave-one-out method for dividing the 10-fold division of CVS3 into each target. The prediction accuracy (i.e., AUC and AUPR) of NRLMF*β* improved significantly compared with that of NRLMF because the score of the interaction pairs in which the value of feature quantities *γ* is 0 was improved. In other words, the AUC and AUPR values increased as the score of interaction pairs with less than 0.5 in NRLMF was selectively increased by rescoring. This indicates that the prediction accuracy was improved by not excessively lowering the score of the pair with less information on interaction (i.e., the feature amount *γ* is small). Fig. 4 represents a histogram for each score in Fig. 3, indicating that the distribution of the score of NRLMF*β* changes for NRLMF. In particular, in NRLMF, the frequency of interaction pairs with a score of 0.1 or less was found to decrease in NRLMF*β*.

### 4.5 Conclusion

Since the score of NRLMF becomes low when a pair of drugs and proteins with less interaction information is used, we proposed a resort algorithm of NRLMF*β*. In NRLMF*β*, the shape of the beta distribution was determined based on the value of the feature quantity *γ* representing the degree of interaction information defined by the expression (13) and the value of the score of NRLMF. The score of NRLMF was recalculated by defining the expected value of the defined beta distribution as the score of NRLMF*β*. In the evaluation experiment, in order to compare the generalization performance of NRLMF and NRLMF*β*, we performed three cross-validations CVS1, CVS2, and CVS3 on four datasets of Nuclear receptor, GPCR, Ion channel, and Enzyme and calculated the average values of AUC and AUPR. Hence, we confirmed that the prediction accuracy of NRLMF*β* was significantly improved compared with that of NRLMF. Thus, we concluded that NRLMF*β* improves the prediction accuracy for drug–target pairs with less interaction information. Future studies need to focus on the number of interactions handled within the prior distribution on the latent feature matrix U,V. We expect that estimating more appropriate parameters and improving prediction accuracy are possible.

## Author contributions

T.B. designed the computational method and performed the experiments. T.B. and M.O. analyzed the results. T.B., M.O., and Y.A. drafted the manuscript. Y.A. supervised the study. All authors are in agreement with the content of the manuscript for publication.

## Competing statement

The authors declare no conflict of interest.
