# Supplementary material for: NRLMFβ: Beta-distribution-rescored neighborhood regularized logistic matrix factorization for improving the performance of drug–target interaction prediction
Source: Biochem Biophys Rep. 2019 Feb 7;18:100615. doi: 10.1016/j.bbrep.2019.01.008 (PMC6370585; doi:10.1016/j.bbrep.2019.01.008)
Supplement: Multimedia component 1 [file mmc1.pdf]

Supplementary Information

NRLMFβ: Beta-distribution-rescored neighborhood regularized logistic matrix factorization for improving the performance of drug–target interaction prediction

Tomohiro Ban[1,2], Masahito Ohue[1,3], Yutaka Akiyama[1,3,4*]

1. School of Computing, Tokyo Institute of Technology, 2-12-1 W8-76 Ookayama, Meguro-ku, Tokyo 152-8550, Japan
2. AIST-TokyoTech Real World Big-Data Computation Open Innovation Laboratory (RWBC-OIL), National Institute of Advanced Industrial Science and Technology, 1-1-1 Umezono, Tsukuba, Ibaraki 305-8560, Japan
3. Middle-Molecule IT-based Drug Discovery Laboratory (MIDL), Tokyo Institute of Technology, RGBT2-A-1C 3-25-10 Tonomachi, Kawasaki-ku, Kawasaki City, Kanagawa 210-0821, Japan
4. Molecular Profiling Research Center for Drug Discovery (molprof), National Institute of Advanced Industrial Science and Technology, 2-4-7 Aomi, Koto-ku, Tokyo 135-0064, Japan

* Corresponding author: akiyama@c.titech.ac.jp

S1 Fixed hyperparameters

Table S1 shows the optimal hyperparameters of NRLMFβ under $\eta_1 = 7, \eta_2 = 3$ determined by grid search. The column 'Dataset' indicates the names of target proteins included in the benchmark dataset. In addition, the column 'CVS' indicates the types of cross-validation scenario for evaluating the performance of drug–target prediction. Conversely, the columns c, K_1, K_2, r, λ_d, λ_t, α, β, and θ indicate the hyperparameters of NRLMFβ. These values were used as fixed hyperparameters to determine an optimal range of the hyperparameters η_1, η_2.

Table S1. Optimal hyperparameters of NRLMFβ under $\eta_1 = 7, \eta_2 = 3$

Dataset	CVS	c	K_1	K_2	r	λ_d	λ_t	α	β	θ
Nuclear receptor	CVS1	5	5	5	50	1.0	1.0	1.0	0.5	0.25
Nuclear receptor	CVS2	5	5	5	50	1.0	1.0	1.0	1.0	0.25
Nuclear receptor	CVS3	5	5	5	50	0.125	0.125	1.0	1.0	0.125
GPCR	CVS1	5	5	5	100	2.0	2.0	4.0	1.0	0.125
GPCR	CVS2	5	5	5	100	2.0	2.0	4.0	1.0	1.0
GPCR	CVS3	5	5	5	100	2.0	2.0	4.0	1.0	1.0
Ion channel	CVS1	5	5	5	50	2.0	2.0	4.0	1.0	0.125
Ion channel	CVS2	5	5	5	50	2.0	2.0	4.0	1.0	0.25
Ion channel	CVS3	5	5	5	100	2.0	2.0	4.0	1.0	0.25
Enzyme	CVS1	5	5	5	100	2.0	2.0	4.0	1.0	0.125
Enzyme	CVS2	5	5	5	50	2.0	2.0	4.0	1.0	0.25
Enzyme	CVS3	5	5	5	100	2.0	2.0	4.0	1.0	0.5

The hyperparameter max_iter of NRLMFβ was set to 100 in all of the above.

S2 Averaged heatmap

Figure S1 shows a heatmap that was generated by merging the heatmaps showed in Figure 2 of the original paper. The values in the heatmap indicate an average of the AUPR for each heatmap. The η_1, η_2 are the hyperparameters of NRLFMβ, and the ranges are from 2 to 512. The frame shows the range of hyperparameters η_1, η_2 whose internal sum is the maximum. Thus, the ranges of hyperparameters are determined as $\eta_1 = \{32, 64, ..., 512\}$ and $\eta_2 = \{4, 8, ..., 32\}$.

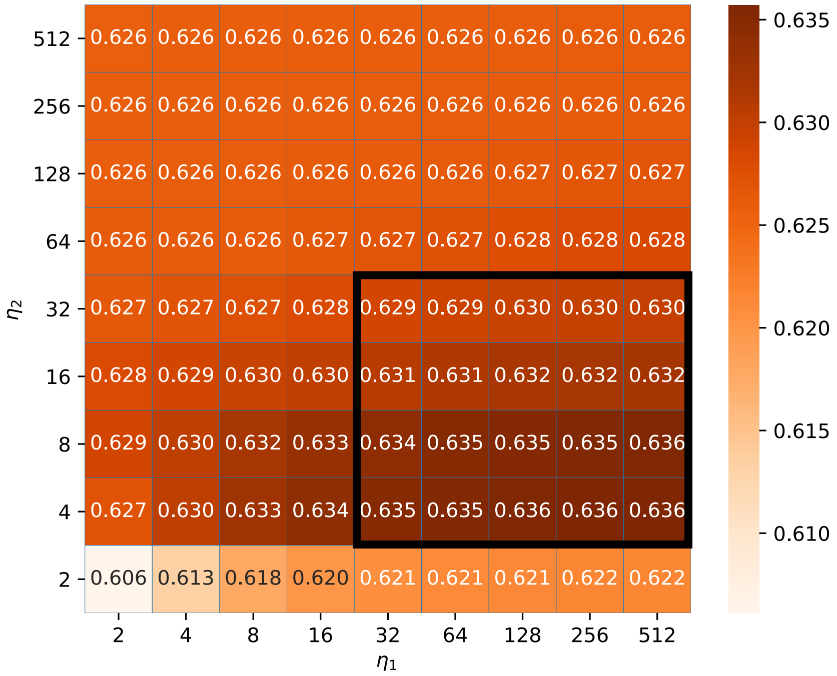

Figure S1. The averaged heatmap of the hyperparameters η_1, η_2 under AUPR scoring.
